# The effects of low frequency electrical stimulation on satellite cell activity in rat skeletal muscle during hindlimb suspension

**DOI:** 10.1186/1471-2121-11-87

**Published:** 2010-11-18

**Authors:** Bao-Ting Zhang, Simon S Yeung, Yue Liu, Hong-Hui Wang, Yu-Min Wan, Shu-Kuan Ling, Hong-Yu Zhang, Ying-Hui Li, Ella W Yeung

**Affiliations:** 1 Muscle Physiology Laboratory, Department of Rehabilitation Sciences, Hong Kong Polytechnic University, Hung Hom, Kowloon, Hong Kong; 2 Space Cell and Molecular Biology Laboratory, Astronaut Research and Training Centre of China, Beijing, PRChina

## Abstract

**Background:**

The ability of skeletal muscle to grow and regenerate is dependent on resident stem cells called satellite cells. It has been shown that chronic hindlimb unloading downregulates the satellite cell activity. This study investigated the role of low-frequency electrical stimulation on satellite cell activity during a 28 d hindlimb suspension in rats.

**Results:**

Mechanical unloading resulted in a 44% reduction in the myofiber cross-sectional area as well as a 29% and 34% reduction in the number of myonuclei and myonuclear domains, respectively, in the soleus muscles (*P *< 0.001 *vs *the weight-bearing control). The number of quiescent (M-cadherin^+^), proliferating (BrdU^+ ^and myoD^+^), and differentiated (myogenin^+^) satellite cells was also reduced by 48-57% compared to the weight-bearing animals (*P *< 0.01 for all). Daily application of electrical stimulation (2 × 3 h at a 20 Hz frequency) partially attenuated the reduction of the fiber cross-sectional area, satellite cell activity, and myonuclear domain (*P *< 0.05 for all). Extensor digitorum longus muscles were not significantly altered by hindlimb unloading.

**Conclusion:**

This study shows that electrical stimulation partially attenuated the decrease in muscle size and satellite cells during hindlimb unloading. The causal relationship between satellite cell activation and electrical stimulation remain to be established.

## Background

Skeletal muscle has a high plasticity for morphological and functional adaptation. The number of myonuclei and size of individual muscle fibers increase rapidly during postnatal growth [1] and with mechanical overload [2]. In contrast, muscle atrophy and the loss of myonuclei are induced with decreased mechanical loading after spaceflight, immobilization, bedrest, and/or inactivity [3-5]. This leads to a decline in peak force, reduced fatigue tolerance, and functional capacity [6].

Under physiological conditions, the capacity of skeletal muscle to regenerate is dependent on resident satellite cells. Satellite cells have been shown to serve as a major source of new myonuclei during muscle regeneration [7] and functional overload [8]. Myonuclear accretion occurs through the incorporation of satellite cell nuclei into the growing myofibers [9]. In contrast, muscle atrophy in response to mechanical unloading is accompanied by a decrease in satellite cell number and mitotic activity [10,11]. The decreased capacity of satellite cells to proliferate is associated with suppressed myonuclear accretion [12] and diminished regenerative potential [13]. Conversely, satellite cell mitotic activity is restored upon mechanical reloading [11,12]. Thus, it seems that impaired satellite cell function contributes to atrophic changes, and the regulation of satellite cell status closely relates to the level of mechanical loading.

The influence of electrical stimulation (ES) in counteracting muscle atrophy has been described in various clinical conditions such as disuse [14], aging [15], spinal cord injury [16], and space flight [17]. Because the atrophic response to disuse is usually greater in the slow-twitch soleus muscles [6,10,18], low frequency (2-20 Hz) pattern of stimulation that matches the muscle properties and the motor unit firing pattern has been shown to be effective in preventing disuse muscle atrophy [14]. Furthermore, chronic low frequency ES-induced proliferation and differentiation of satellite cells in hypothyroid and aging muscles have been reported [15,19]. However, the characterization of muscle satellite cell responses in the context of low frequency electrical stimulation in mechanically unloaded conditions has not been fully clarified. The present study was performed to investigate the effects of low frequency ES (20 Hz, 6 h·day^-1^) on satellite cell activity in the slow-twitch soleus and fast-twitch extensor digitorum longus (EDL) muscles during hindlimb suspension. Here, we show that 28 d of mechanical unloading leads to a reduced number of quiescent, proliferating, and differentiated satellite cells in the soleus muscle. Impairments in satellite cell activity in the unloaded soleus muscle were partially attenuated by low frequency ES.

## Methods

### Animals

Male Wistar rats (4-5 wk old, body weight 170 ± 20 g) were used in this study. Animal care procedures and the experimental protocol were approved by the Animal Ethics Committee of The Hong Kong Polytechnic University.

### Hindlimb suspension procedure

The animals were suspended from the hindlimb for a period of 28 d. The hindlimb suspension procedure described by Morey-Holton and Globus [20] was adopted for this study. Briefly, a strip of adhesive tape (15 cm × 0.5 cm) was applied to the animal's tail, which was suspended by passing the tape through a fish-line swivel that was attached to a metal bar on the top of the cage. This allowed the forelimbs to have contact with the grid floor and allowed the animals to move around the cage for free access to food and water. The suspension height was adjusted to prevent the hindlimbs from touching any supporting surface while maintaining a suspension angle of approximately 30°. The animal's overall appearance, drinking and eating habits, and tail were monitored four times per day. The distal tip of the tail was examined to ensure that the procedure did not occlude blood flow to the tail (i.e., the tail remained pink). The body weight was measured weekly with a suspension apparatus so that the hindlimbs remained suspended while the animals were weighed.

### Electrical stimulation

Age-matched animals were randomly assigned to either the weight-bearing control (WB) or hindlimb unloading (HU) group (*n *= 10 each). During unloading, the muscles of one hindlimb were subjected to electrical stimulation (HU-ES) and the contralateral leg served as the control (HU). The selection of the stimulated leg was randomized.

To achieve electrical stimulation on the unloaded limb, rats were anaesthetized with sodium pentobarbital (40 mg kg^-1 ^I.P.). The hindlimb to be stimulated was shaved and two electrodes (0.5 cm × 2 cm) were placed on the skin overlying the middle of the calf (plantarflexors) and the lateral side of the tibia (dorsiflexors). Insulating material was placed between the two electrodes to prevent the formation of a short circuit. Both electrodes were secured in place with ventilated tape. Stimuli (0.2 ms pulse duration, 20 Hz) were applied twice a day for 3 h (with a 2 h rest between treatments) for a total of 6 h of treatment per day using a DC-driven electrical stimulator (CEFAR Digital Unit, Lund, Sweden) during the hindlimb suspension period. The stimulation intensity was set to induce visible muscle contractions. Since a decrease in the conductivity between the electrodes and the skin could occur due to hair growth or loose electrodes, the threshold of stimulation intensity (range from 0.5 - 2 mA) was modified daily to ensure that tetanic contraction of the muscles could be induced. This mode of electrical stimulation induced simultaneous isometric contractions of the dorsiflexors and plantarflexors, and thus maintained the ankle joint static during the stimulation.

### Histology and immunohistochemistry

At the end of the suspension, the rats were anaesthetized with sodium pentobarbital (40 mg kg^-1 ^I.P.) and killed by cervical dislocation. The dissected soleus and EDL muscles from each limb were snap frozen in liquid nitrogen-cooled isopentane and then embedded in OCT medium. Serial cross-sections (10 μm thickness) were cut from the mid-belly of the muscles on a cryostat at -20°C for histological and immunohistochemical staining. Hematoxylin and eosin (H&E) staining was performed on sections to examine the general morphology and to determine the cross-sectional area (CSA) of the fiber.

Immunohistochemical staining was performed to detect the markers of quiescent (M-cadherin), proliferating (BrdU and myoD), and differentiated (myogenin) satellite cells [11,12,15,21,22]. To determine satellite cell proliferation *in vivo *at a given time point, BrdU labeling was used. Rats were injected with BrdU intraperitoneally (Sigma, St. Louis, MO, USA, 45 mg kg^-1^) 1 h before they were killed.

For immunostaining, the cryosections were fixed in cold acetone (4°C) for 10 min and then incubated for 30 min in a 1% bovine serum albumin (BSA)/PBS solution to block non-specific binding. For BrdU staining, the cryosections were denatured with 2 N HCL in PBS for 30 min at 37°C prior to serum blocking. The sections were then incubated overnight at 4°C with rabbit polyclonal anti-M-cadherin (H-71, 1:50 dilution; Santa Cruz Biotechnology, Santa Cruz, CA, USA), mouse monoclonal anti-BrdU (BU-33, 1:50 dilution; Sigma), mouse monoclonal anti-myoD (5.8A, 1:50 dilution; Santa Cruz Biotechnology), or mouse monoclonal anti-myogenin (F12B, 1:100 dilution; Sigma) together with a goat polyclonal anti-dystrophin antibody (C-terminus, 1:50 dilution; Santa Cruz Biotechnology) for double staining. Following three washes in PBS, the sections were incubated with FITC-conjugated donkey anti-mouse or anti-rabbit IgG F(ab')_2 _fragments (1:200 dilution; Jackson ImmunoResearch Laboratories, West Grove, PA, USA) and TRITC-conjugated donkey anti-goat IgG F(ab')_2 _fragments (1:200 dilution; Jackson ImmunoResearch Laboratories) for 1 h. The sections were again washed in PBS, stained with the nuclear dye 4',6-diamidino-2-phenylindole dihydrochloride (DAPI; 0.25 μg ml^-1^; Roche Diagnostics Corporation, Indianapolis, IN, USA) for 10 min, washed, and mounted in Mowiol mounting medium (Merck KGaA, Darmstadt, Germany).

The images were captured using a laser confocal microscope (Carl Zeiss LSM 510 Meta system, Oberkochen, Germany) with differential interference contrast (DIC) optics. All quantifications were performed in a blinded fashion.

### Determination of fiber cross-sectional area

Digital images (4×) of H&E stained muscle sections were captured and the cross-sectional area for the muscles was determined from the composite images of the entire muscle cross-section. With the aid of an image morphometry program (ImageJ 1.32j, NIH, Bethesda, MD, USA), the outline of the individual fibers was traced and the total number of fibers was assessed. The area was calculated and expressed in μm^2^.

### Determination of satellite cell number in muscle fiber

Cells labeled for M-cadherin and nuclei labeled for BrdU, myoD or myogenin were quantified in two sections for each muscle. The total number of positive cells or nuclei was counted and expressed per 100 muscle fibers.

### Determination of myonuclear number and domain

The myonuclear number and domain was determined on the cross-section that was immunostained with dystrophin and DAPI. The number of DAPI-labeled myonuclei was determined on sections stained for dystrophin from at least 50 fibers and expressed as the myonuclear number per fiber cross-section. Only the nuclei located within the sarcolemmal boundaries defined by dystrophin staining were included. The nuclei included both existing myonuclei and satellite cells that had divided and already entered the muscle fiber.

The number of myonuclei per mm of fiber (X) was calculated based on the formula: X = (NL)/(d + I), where N is the number of myonuclei in a cross-section of a fiber, L is the fiber segment length (accepted as 1 mm), d is the thickness of the cryostat cut cross-section (10 μm), and I is the myonuclei length of 14.3 μm for rat soleus and EDL muscles [23,24]. The myonuclear domain (cytoplasmic myofiber volume/myonucleus) (Y) was calculated using the formula: Y = (CL)/X, where C is the CSA of the muscle fiber, L is the segment length (1 mm), and X is the number of myonuclei per mm of myofiber segment.

### Statistics

The Shapiro-Wilk statistic was performed for each dependent variable to ensure that a normality in distribution existed. For normally distributed data (CSA, myonuclear number and domain), one-way ANOVA with Bonferroni *post hoc *analysis were used to compare the differences between the WB, HU, and HU-ES groups. If data exhibited a non-normal distribution (satellite cell markers), a non-parametric Kruskal-Wallis with Mann-Whitney U statistics as a *post hoc *measure was used to detect the differences between the groups. The values were expressed as means ± SEM. The level of significance was set at P < 0.05.

## Results

### Body mass

The body mass at baseline was similar in both groups. At the end of 28 d, the hindlimb unloaded animals gained less body mass compared to the age-matched, weight-bearing animals (248.4 ± 10.2 g vs. 355.1 ± 9.0 g, respectively; *P *< 0.05). This difference could have been caused by the reduction in the overall growth rate, particularly during the first two weeks of the suspension [12].

### Fiber cross-sectional area

**Figure 1:**
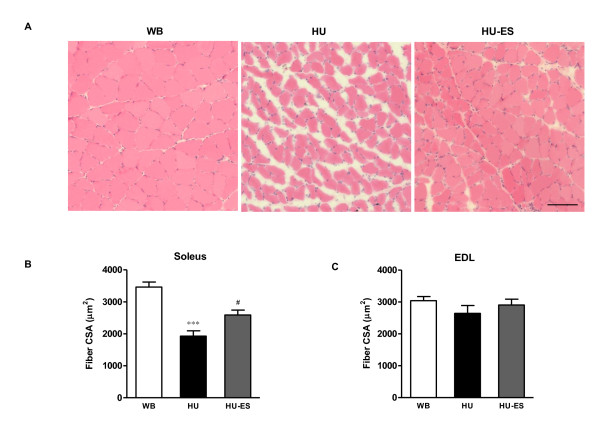
**Muscle fiber size**. **(A) **Cross-sections of soleus muscle stained with H & E from WB, HU, and HU-ES groups. Scale bar = 100 μm. Mean fiber cross-sectional area (μm^2^) of soleus **(B) **and EDL (**C) **muscles in the WB, HU, and HU-ES groups. *** *P *< 0.001, significant difference between WB and HU groups; ^# ^*P *< 0.05, significant difference between HU and HU-ES groups. *n *= 4. Data are means ± SEM.

The CSA measurements of the muscle fiber size were used as a best measure of muscle atrophy to avoid potential confounding factors related to extracellular space changes. Soleus and EDL muscle sections from the WB, HU, and HU-ES groups were stained for H&E in order to assess morphology and to measure the fiber CSA. The mean CSA of the soleus muscle in the HU group was 44% lower than that of the control group (1926 ± 168 μm^2 ^vs. 3463 ± 155 μm^2^, respectively; *P *< 0.001). There was an attenuation of fiber atrophy in the HU-ES group (2589 ± 153 μm^2^, *P *< 0.05) compared to the untreated HU muscles (Figure 1). There were no statistically significant differences in mean fiber CSA between WB and HU or between HU and HU-ES in the EDL muscles.

### Myonuclear number and domain size

**Figure 2:**
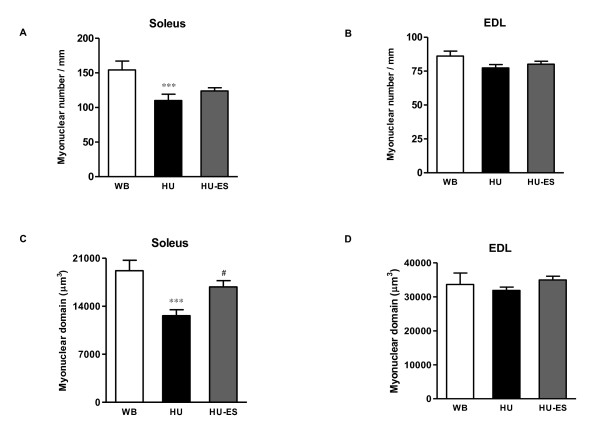
**Myonuclear number and domain size**. **(A, B) **Numbers of myonuclei per millimeter fiber length, and **(C, D) **Myonuclear domain size for WB, HU, and HU-ES groups. *** *P *< 0.001, significant difference between WB and HU groups; ^# ^*P *< 0.05, significant difference between HU and HU-ES groups. *n *= 4. Data are means ± SEM.

The myonuclear number was assessed by determining the number of DAPI-stained nuclei located within the dystrophin-positive sarcolemma. The values obtained were in agreement with previous studies on single fiber explants [11,12]. A 29% lower myonuclei number was observed in the HU group when compared to the control group (110 ± 9 vs 154 ± 13, respectively; *P *< 0.001) (Figure 2A). However, ES failed to restore the myonuclear number during unloading in the soleus muscles (HU-ES: 124 ± 7).

Unloading also resulted in a significantly lower myonuclear domain size (34%) in the unloaded soleus muscle (HU soleus: 12622 ± 881 μm^3^, WB soleus: 19192 ± 1516 μm^3^, *P *< 0.001) (Figure 2C). Electrical stimulation partially attenuated the loss in the myonuclear domain in the unloaded soleus muscle (16818 ± 915 μm^3^, *P *< 0.05). The myonuclear number and domain were not significantly different in the EDL muscles of the WB, HU, and HU-ES groups (*P >*0.05) (Figure 2B and 2D).

### Satellite cells

**Figure 3:**
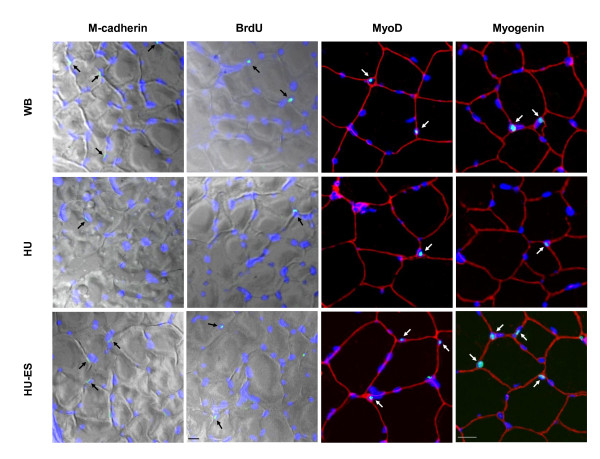
**Changes in the markers of satellite cells**. Representative cross-sections of soleus muscle immunostained for M-cadherin, BrdU, myoD, and myogenin (green, *arrows*), respectively. The two columns on the left are DIC images. The two columns on the right show dystrophin immunolabeling (red) to identify fiber profiles and nuclear staining with DAPI (blue). Scale bar = 20 μm.

To investigate changes in the satellite cell activity, we immunostained cross-sections of soleus and EDL muscles for markers of quiescent (M-cadherin), proliferating (BrdU and myoD), and differentiated (myogenin) satellite cells. The number of M-cadherin^+^, BrdU^+^, myoD^+^, and myogenin^+ ^satellite cells was quantified in the WB, HU, and HU-ES groups. Positive staining for M-cadherin extended closely along the sarcolemma, which was consistent with its transmembrane function. BrdU was detected in nuclei during cell division. In addition, the transcription factors myoD and myogenin were expressed in nuclei and were present during satellite cell proliferation and differentiation (Figure 3).

**Figure 4:**
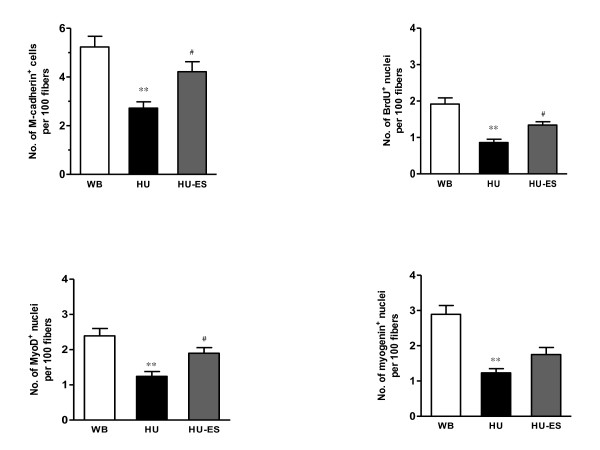
**Quantification of satellite cells labeled positive in soleus muscles**. Number of M-cadherin^+ ^cells or BrdU^+^, myoD^+^, and myogenin^+ ^nuclei per 100 fibers in cross sections of the soleus muscles. ** *P *< 0.01, significant difference between WB and HU groups; ^# ^*P *< 0.05, significant difference between HU and HU-ES groups. *n *= 6. Data are means ± SEM.

After 28 d of unloading, the number of quiescent (M-cadherin^+^), proliferating (BrdU^+ ^and myoD^+^), and differentiated (myogenin^+^) satellite cells per 100 fibers in the soleus muscle was 48%, 55%, 48%, and 57% lower, respectively, compared to the WB muscles (*P *< 0.01 for all markers) (Figure 4). This unloading-associated impairment in the satellite cell activity was partially attenuated by the application of ES during unloading by 55% for M-cadherin, 56% for BrdU, 53% for myoD (*P *< 0.05 for all three markers), and 42% for myogenin (*P *> 0.05) compared to the untreated muscles (Figure 4).

No statistical differences in the number of M-cadherin^+^, BrdU^+^, myoD^+ ^and myogenin^+ ^satellite cells were evident in the EDL muscles of the WB, HU, and HU-ES groups (data not shown).

## Discussion

The effects of electrical stimulation on the satellite cell activity in muscles undergoing disuse-induced atrophy were determined using morphological and immunohistochemical analyses. Our hypothesis was that the daily application of electrical stimulation would attenuate muscle atrophy, particularly in the slow-twitch soleus muscles, by enhancing satellite cell activity. The major findings were that (i) the loss of muscle CSA and myonuclear domain in the soleus muscle were attenuated by the application of electrical stimulation; and (ii) the number of quiescent (M-cadherin^+^), and proliferating (BrdU^+ ^and myoD^+^) satellite cells were higher in the electrically stimulated, unloaded soleus.

Consistent with previous studies [6,10,18], slow-twitch soleus muscle showed greater atrophy than fast-twitch EDL muscles following unloading. Slow muscle plays a strong role in antigravity function and is highly dependent on gravity for the normal expression of protein mass and slow phenotype. Conversely, fast-twitch EDL muscle is physically active and does not have an antigravity function. The fiber-type specific atrophic responses can be attributed to many factors such as differences in neuronal recruitment pattern, fiber diameter, SR calcium release function [6] and the recently reported differences in the levels of capillarisation [25]. We analyzed the CSA of the soleus and EDL muscle fibers in the age-matched WB, HU, and HU-ES groups to ascertain the effects of electrical stimulation on muscle fiber size. The mean CSA of the soleus muscle in the HU-ES group was 34% larger than that of the HU group after 28 d of unloading. ES has no significant influence on the muscle size of the unloaded EDL muscles. This may be because the fiber atrophy was very mild in the EDL muscle. Furthermore, the pattern of stimulation resembles the motor unit firing frequency of slow-twitch muscle fibers. Whether a higher stimulation frequency would benefit the EDL muscle fibers need to be examined in future studies.

In this study, we used a panel of antibodies that recognize proteins uniquely expressed in satellite cells and their progeny during the quiescent, proliferating, or differentiating stage. M-cadherin, a calcium-dependent transmembrane glycoprotein, anchors satellite cells to the sarcolemma and is regarded as one of the markers for quiescent satellite cells [21,22]. We also examined mitotically active satellite cells upon electrical stimulation during unloading, using antibodies specific for BrdU and myoD that identify the status of satellite cell proliferation [19,22]. ES induced a higher number of quiescent and proliferating satellite cells in the unloaded soleus muscles. Further, there was a trend for higher number of satellite cells positively stained for myogenin, a marker of myogenic differentiation [15,22], in the stimulated soleus muscles, although the difference did not reach statistical significance. Satellite cells proliferate to provide myonuclei for growing myofibers. We have conducted this study in growing animals due to the higher mitotic activity and greater abundance of satellite cells [10,12]. It constitutes a good model in which to study the influence of different interventions on satellite cell proliferation and differentiation since they are less detectable in weight stable animals. Our findings agree with previous studies [10,11,26] that have shown that unloading results in a dramatic loss in the satellite cell number and mitotic activity in growing muscles. This suggests that the loss of satellite cells was closely related to a decrease in mechanical load. The decline in satellite cell activity resulted in impaired muscle growth capacity. In fact, the reduction in the number of quiescent and mitotically active satellite cells may reflect activation of the apoptotic process during mechanical unloading [27]. In contrast, electrical stimulation of hindlimb-unloaded soleus muscles maintained the satellite cell mitotic activity to some extent. Although electrical stimulation provides some protection against muscle atrophy by influencing the satellite cell activity, the results showed only a modest benefit, since the reduction in the number of satellite cells was not restored to similar levels in the WB control conditions. In this study, we chose to apply the electrical stimulation at a frequency of 20 Hz, because this protocol resembles the motor unit firing frequency of slow-twitch muscle fibers [28]. Future studies are needed to optimize the electrical stimulation parameters and the optimal duration in order to achieve the desired responses.

Satellite cell proliferation and incorporation are closely associated with changes in the myonuclear number [22]. It has been reported that the number of myonuclei decreases in muscles undergoing atrophy in several different conditions, such as spinal cord injury, microgravity, and hindlimb suspension [3,29,30]. In this study, we observed that the myonuclear number and domain were lower (-29% and -34%, respectively) in the soleus muscle fibers following HU when compared to the age-matched WB muscles. The HU-induced loss of myonuclei in the adult rat muscle has been suggested to be a consequence of apoptosis [27]. However, a previous study [12] suggested that in the unloaded, growing rat muscles, inhibition of myonuclear accretion, and not apoptosis-related loss of myonuclei, was the major cause of the smaller number of myonuclei.

Although electrical stimulation during 28 d of unloading induced a higher number (~50%) of quiescent and proliferating satellite cells in the soleus muscle, there was no significant increase in the number of myonuclei. These results indicate that the activated satellite cells from electrical stimulation were insufficient to restore the myonuclear loss during unloading. There are several points to consider for these observations. First, these results may be associated with an inability of satellite cells to incorporate into the muscle fibers. A previous study showed that satellite cell adhesion was inhibited by mechanical unloading, this may inhibit myonuclear accretion [12]. Second, the stimulation parameters used was not effective enough to induce a significant increase in terminally differentiated satellite cells, these parameters may need to be further modified in order to achieve a more optimized effect. In this study, we observed a significantly larger myonuclear domain in the HU-ES soleus muscles. Since the whole protein-to-DNA ratio in muscle has been accepted as a measure of the myonuclear domain [31], increased protein content can be accomplished by increasing the myonuclear domain of each myonucleus without changing the total number of myonuclei. As such, a third possibility is that electrical stimulation plays a role in cellular protein metabolism, rather than through satellite cell-related pathways. In fact, the positive effects of electrical stimulation on protein synthesis [32] and degradation [16] have been reported in several conditions that lead to atrophy. While a causal relationship cannot be inferred from the present data, the mechanism underlying the recruitment of satellite cells with electrical stimulation in preventing muscle atrophy may be achieved from experiments in which the satellite cells are ablated.

Reduction in satellite cell activity was not observed in unloaded EDL muscles. These data may be associated with an unequal distribution of satellite cells in different fiber types where a higher number of satellite cells is associated with slow-twitch muscles [33]. This may indicate that a larger number of satellite cells is required to maintain the muscle growth and repair in the slow-twitch muscles. We did not determine muscle fiber types in this study, and further investigation is required to elucidate the muscle fiber type and specific adaptation of satellite cells with electrical stimulation.

One of the limitations of the study was the use of M-cadherin staining to quantify satellite cell numbers, which could have led us to underestimate the absolute number. Pax7 staining is used to determine the satellite cell pool size [34]. Detection of Pax7^+ ^satellite cells and/or co-localization of Pax7 with myoD or myogenin may provide more valuable information on the overall satellite cell number regardless of the activation state. Another possible limitation was the use of surface electrodes to deliver electrical currents to the muscles, in contrast to surgically-implanted electrodes, which has been used in other studies [14-16,19]. However, this is an important point to consider, since satellite cells would be activated in response to injury [35]. Furthermore, the use of surface electrodes is compatible with future research and application in human subjects. To ensure that electrical currents (2 × 3 h day^-1^) were efficiently delivered to the muscles, we shaved the hindlimb of the animals and replaced the electrodes every 2-3 d in order to prevent resistance caused by hair growth and to ensure that the electrodes were well-attached. The general morphology and satellite cell properties differed in stimulated and contralateral unstimulated muscles following unloading, suggesting that the electrical currents were successfully delivered to the target muscles and had little influence on the contralateral unstimulated muscles.

Several studies have demonstrated that chronic, low-frequency electrical stimulation results in skeletal muscle angiogenesis through the up-regulation of hepatocyte growth factor (HGF) and vascular endothelial growth factor (VEGF) [36,37]. In addition, it has been shown that vessel-related progenitors possess high myogenic potential and may provide a secondary source of myogenesis as well as contribute to neoangiogenesis [38]. It is therefore possible that electrical stimulation induces angiogenesis in the muscles that promote myogenic regeneration and the recovery of lost muscle mass. A recent study demonstrated that the dual delivery of VEGF and insulin-like growth factor-1 (IGF1) led to parallel angiogenesis, myogenesis with activation and proliferation of satellite cells, and a decrease in cell apoptosis [39]. The possible role of electrical stimulation in the regulation of spatial and temporal patterns of myogenic and angiogenic events remains to be established.

Although muscle atrophy was not fully recovered with electrical stimulation in this study, we did observe some promising results that electrical stimulation can induce the proliferation and activation of satellite cells. A variety of strategies have previously been used to countermeasure muscle atrophy, but no strategy has yet to completely prevent the atrophic response. Electrical stimulation may possibly serve as a valuable adjunct to facilitate the recovery of muscle atrophy. It also has a practical implication in clinical situations, where it is either difficult or impossible for patients to return to weight-bearing conditions.

## Conclusion

This study has demonstrated that mechanical unloading impairs the satellite cell activity in slow-twitch soleus muscles. The application of low-frequency electrical stimulation partially attenuated muscle atrophy and satellite cell inactivity. The role of satellite cells in the prevention of unloading-induced muscle atrophy by ES needs further exploration.

## Authors' contributions

EWY, SSY, and YHL conceived and designed the experiments. YL, HHW, YMW, SKL, and HYZ performed the unloading experiments, muscle isolation, and assisted with some of the immunostaining studies. BTZ performed immunostaining, imaging, and helped to draft the manuscript. EWY and SSY analyzed the data and wrote the manuscript. All authors read and approved the final manuscript.
